# Genetic Variants of Complement Factor H Y402H (rs1061170), C2 R102G (rs2230199), and C3 E318D (rs9332739) and Response to Intravitreal Anti-VEGF Treatment in Patients with Exudative Age-Related Macular Degeneration

**DOI:** 10.3390/medicina58050658

**Published:** 2022-05-13

**Authors:** Agnieszka Kubicka-Trząska, Katarzyna Żuber-Łaskawiec, Sylwia Dziedzina, Marek Sanak, Bożena Romanowska-Dixon, Izabella Karska-Basta

**Affiliations:** 1 Clinic of Ophtalmology and Ocular Oncology, Department of Ophtalmology, Faculty of Medicine, Jagiellonian University Medical College, 31-501 Krakow, Poland; katarzyna.zuber-laskawiec@uj.edu.pl (K.Ż.-Ł.); bozena.romanowska-dixon@uj.edu.pl (B.R.-D.); izabella.karska-basta@uj.edu.pl (I.K.-B.); 2 Molecular Biology and Clinical Genetics Unit, Department of Internal Medicine, Faculty of Medicine, Jagiellonian University Medical College, 31-501 Krakow, Poland; sylwia.dziedzina@uj.edu.pl (S.D.); marek.sanak@uj.edu.pl (M.S.)

**Keywords:** complement system, single nucleotide polymorphism, age-related macular degeneration, anti-VEGF therapy, switching therapy

## Abstract

*Background and Objectives*: To assess the association between the single nucleotide polymorphisms (SNPs) in the genes encoding complement factors CFH, C2, and C3 (Y402H rs1061170, R102G rs2230199, and E318D rs9332739, respectively) and response to intravitreal anti-vascular endothelial growth factor (VEGF) therapy in patients with exudative age-related macular degeneration (AMD). *Materials and Methods*: The study included 111 patients with exudative AMD treated with intravitreal bevacizumab or ranibizumab injections. Response to therapy was assessed on the basis of best-corrected visual acuity (BCVA) and central retinal thickness (CRT) measured every 4 weeks for 12 months. The control group included 58 individuals without AMD. The SNPs were genotyped by a real-time polymerase chain reaction in genomic DNA isolated from peripheral blood samples. *Results*: The CC genotype in SNP rs1061170 of the *CFH* gene was more frequent in patients with AMD than in controls (*p* = 0.0058). It was also more common among the 28 patients (25.2%) with poor response to therapy compared with good responders (*p* = 0.0002). Poor responders, especially those without this genotype, benefited from switching to another anti-VEGF drug. At the last follow-up assessment, carriers of this genotype had significantly worse BCVA (*p* = 0.0350) and greater CRT (*p* = 0.0168) than noncarriers. TT genotype carriers showed improved BCVA (*p* = 0.0467) and reduced CRT compared with CC and CT genotype carriers (*p* = 0.0194). No associations with AMD or anti-VEGF therapy outcomes for SNP rs9332739 in the *C2* gene and SNP rs2230199 in the *C3* gene were found. *Conclusions*: The CC genotype for SNP rs1061170 in the *CFH* gene was associated with AMD in our population. Additionally, it promoted a poor response to anti-VEGF therapy. On the other hand, TT genotype carriers showed better functional and anatomical response to anti-VEGF therapy at 12 months than carriers of the other genotypes for this SNP.

## 1. Introduction

Age-related macular degeneration (AMD) is the most common cause of central blindness in the elderly population in developed countries [1]. The disease affects approximately 30 to 50 million people, and its prevalence is expected to double by 2050 [2,3]. The characteristic feature of AMD is central vision impairment that either occurs gradually due to progressive geographic atrophy (typical for dry AMD) or acutely due to retinal hemorrhage and fluid exudation from choroidal neovascularization (CNV) in exudative AMD [2].

In the last few years, several hypotheses explaining the pathogenesis of AMD have been proposed. However, despite extensive clinical and experimental research, the pathogenetic mechanisms underlying the disease remain unclear. It seems that AMD involves multiple complex processes such as increased oxidative stress, excessive complement activation and inflammatory reactions in the subretinal space (parainflammation), autophagy dysregulation, and an interplay between metabolic, environmental, and genetic factors [4,5,6,7,8,9,10,11,12]. In the past decade, genetic functional studies have revealed new possible mechanisms involved in the pathophysiology of AMD, with the potential to identify molecular targets for novel therapies. Additionally, there has been a growing interest in genetic testing to predict the risk of AMD and response to treatment [13,14,15].

The complement system, and particularly the alternative pathway, has shown a strong association with AMD, and there is evidence that it plays a central role in the etiology of AMD [16,17,18]. The complement system, which is a part of the innate immune system, consists of about 50 proteins circulating in the blood as inactive components. A cleavage event triggers the activation of a protease cascade on the surface of either pathogens or host cells. The result of this activation is the stimulation of phagocytes to clear foreign and damaged material, inflammation, and activation of the cell-killing membrane attack complex [19].

Studies on the several variants of genes encoding complement proteins, including complement factor F (*CFH*), factor I (*CFI*), and factor B (*CFB*) as well as complement components C3, C2, and C9, showed a genetic link between AMD and the complement system [20,21,22,23,24,25,26,27,28,29,30]. Some of these genetic polymorphisms are not only risk factors for the development and progression of the disease but they may be valuable pharmacogenetic predictors of response to antiangiogenic therapy [31,32,33,34,35,36,37,38].

The important role of the complement system in the etiopathogenesis of AMD has been also supported by recent multicenter studies assessing the efficacy of the intravitreal treatment with complement cascade inhibitors targeting complement proteins C3, C5, and CD59, injected either alone or in combination with vascular endothelial growth factor (VEGF) inhibitors, in patients with exudative AMD. Inhibition with bispecific decoy receptor fusion protein that simultaneously binds VEGF, C3b, and C4b is also under investigation [39,40].

Despite the ongoing search for novel therapies, intravitreal anti-VEGF injections remain the gold standard treatment of exudative AMD. However, clinical studies show that not all patients with this type of AMD benefit from this therapy, with about 14% to 30% of patients showing poor response to antiangiogenic drugs. In these patients, progression of macular lesions and worsening of vision is seen despite therapy [41,42,43,44]. This has led to a growing interest in studies on factors and mechanisms underlying the lack of positive response to therapy.

The aim of this study was to assess the prevalence of single nucleotide polymorphisms (SNPs) in the *CFH* (Y402H rs1061170), *C2* (E318D rs9332739), and *C3* (R102G (rs2230199) genes in patients with exudative AMD. Moreover, we determined associations between these SNPs and the risk of AMD and response to intravitreal anti-VEGF therapy. Associations between potential risk factors for AMD (sex, age, smoking, living environment, and a family history of AMD) and the development of AMD irrespective of the genetic polymorphisms were also assessed. Finally, we investigated whether these genetic variants in the complement system affect treatment outcomes after switching from one anti-VEGF drug to another in treatment-resistant patients.

## 2. Materials and Methods

This retrospective case–control study included 111 patients with exudative AMD referred to Retinal Disorders Outpatient Clinic at the Department of Ophthalmology and Ocular Oncology, University Hospital in Krakow, Poland.

The diagnosis of AMD was based on typical fundus findings documented by digital photography (fundus camera Topcon-TRC-50DX, Tokyo, Japan), optical coherence tomography (OCT; Topcon 3D OCT 2000, Tokyo, Japan), fluorescein angiography (FA; Spectralis HRA-OCT, Heidelberg Engineering, Heidelberg, Germany), and, in some cases, on OCT angiography (Topcon, DRI OCT-1, Atlantis, Tokyo, Japan).

All patients underwent comprehensive ophthalmic examination, and AMD was diagnosed on the basis of the classification developed by Ferris et al. [44]. In this standard clinical classification system, fundus lesions are assessed within two-disc diameters of the fovea. Active exudative AMD was diagnosed if CNV or fibrovascular pigment epithelium detachment (PED) with or without subretinal or retinal pigment epithelial (RPE) hemorrhage was present.

On OCT, morphologic parameters indicating disease activity were evaluated, such as intraretinal fluid, subretinal fluid, serous PED, and central retinal thickness (CRT). On FA, classic CNV was identified as an area of a lacy pattern with early staining and progressive leakage in the late frames. Occult CNV was classified as areas of stippled hyperfluorescence that appeared in the mid and late phases. Mixed CNV was detected if both classic and occult CNV lesion components were present. The type and diameter of CNV on FA were assessed. The OCT criteria for CNV included an elevated submacular hyperreflective lesion accompanied by subretinal and/or intraretinal fluid. In all patients, the Amsler grid test was performed, and the best-corrected visual acuity (BCVA) was assessed using the Snellen chart, and then converted into the logMAR scale. Follow-up examinations were performed every 4 weeks for 12 months and included the same procedures as the baseline examination, except for FA and OCTA.

Patients were treated with one of two anti-VEGF drugs: ranibizumab (Lucentis, 0.5 mg/0.05 mL; Novartis, Germany) and bevacizumab (Avastin, 1.25 mg/0.05 mL, Roche, Switzerland). The treatment protocol consisted of the loading phase of therapy, during which patients received 3 monthly injections of an anti-VEGF drug. This was followed by the maintenance therapy, with ranibizumab and bevacizumab injected on an as-needed basis based on the results of a clinical examination performed every 4 weeks. The worsening of BCVA associated with clinical evidence of disease activity assessed on OCT (evidence of intraretinal fluid, subretinal fluid, serous PED, or increased CRT) were indications for injection of an anti-VEGF drug. The choice of the anti-VEGF drug was at the discretion of the treating retina specialist. The decision to use anti-VEGF therapy was based on patient’s medical history. Individuals with a history of recent myocardial infarction or cerebrovascular accident were not considered for intravitreal injections of bevacizumab, because the therapy may be associated with increased systemic risk due to a sustained suppression of systemic VEGF levels.

When patients failed to show clinical improvement within the first 4 to 6 months of initiating anti-VEGF therapy, they were switched to the other anti-VEGF drug (from bevacizumab to ranibizumab or from ranibizumab to bevacizumab).

Poor response to anti-VEGF therapy was defined as follows: No changes in CRT or a reduction in CRT ≤ 10% (anatomical poor responders) or no improvement in BCVA or a deterioration in BCVA by ≥ 1 line on the Snellen chart (functional poor responders). The presence of morphologic parameters on OCT scans, such as persistent or increased intraretinal fluid, persistent or increased subretinal fluid, and an enlarged area of serous PED, also indicated poor response to therapy.

The control group included 58 age- and sex-matched individuals without signs of AMD, who were scheduled for senile cataract surgery. The absence of AMD was determined by clinical examination under dilated fundus examination and using the classification by Ferris et al. [44]. Individuals with no visible drusen or pigmentary abnormalities or with small drusen (<63 μm) were considered to have no signs of AMD. There was no family history of a hereditary disease or cancer either in patients with AMD or in the control group.

The study was conducted in accordance with the ethical standards of the institutional research committee and with the Declaration of Helsinki. The protocol was approved by Jagiellonian University Bioethical Committee (no. KBET/67/B/2013), and all participants provided written informed consent to participate in the study.

### 2.1. Genotyping

#### 2.1.1. Genomic DNA Isolation

Peripheral venous blood samples were collected from all participants, using sodium EDTA as an anticoagulant. Blood leukocytes were obtained by centrifugation of cell-rich plasma. Genomic DNA was isolated from leukocytes by chaotropic lysis (DNAzol, Thermo Fisher Scientific, Waltham, MA, USA). Extracted DNA was stored as an aqueous solution at a temperature of −20 °C until genotyping.

#### 2.1.2. Genotyping of Single Nucleotide Polymorphisms

SNPs were genotyped from genomic DNA by a real-time polymerase chain reaction based on the 5′nuclease assay. Commercial TaqMan assays were acquired for each of the SNPs studied from Thermo Fisher Scientific. In brief, the TaqMan assay uses a universal master mix of reagents, including a thermostable hot-start polymerase and a set of two primers and two fluorogenic genetic probes complementary to each SNP. During the real-time polymerase chain reaction, a region of the genomic DNA encompassing SNP is amplified. Either of the probes or both probes can hybridize with the reaction template and then degrade due to 5′-exonuclease activity of the polymerase. Thus, a fluorescence signal measured during each cycle increases, and due to a spectral difference in fluorophore emission, it can discriminate variations of a single nucleotide. The method is validated by the manufacturer of the HT 7900 thermocycler (Applied Biosystems, Foster City, CA, USA).

### 2.2. Statistical Analysis

The analysis of variance was used for repeatable parameters. As Snellen BCVA results did not show normal distribution after conversion to the BCVA logMAR scale, the Friedmann test was used for statistical analysis. For independent variables, the Mann–Whitney test was used. The Wald χ^2^ analysis was used to test the significance of distribution differences in genotypes and alleles between groups. To determine the strength of the relationship two ranked-ordered variables, the nonparametric Spearman’s correlation coefficient was used.

For the purpose of the statistical analysis, the coding scheme of variables was applied. The dependent variable AMD was coded as follows: control group = 0, AMD group = 1. Independent (explanatory) variables were coded using the following scheme: sex, males = 0, females = 1; age ≤ 60 years = 0, age > 60 years = 1; smoking, never = 0, current/former = 1; living environment, rural = 0, urban = 1; and family history of AMD, negative = 0, positive = 1.

First, we assessed the incidence of AMD depending on factors involved in the pathophysiology of AMD, such as sex, age, smoking, living environment, and a positive family history of AMD. Next, allele and genotype associations between the polymorphisms of complement pathway genes and AMD were evaluated. The number (percentage) of patients was calculated in contingency tables. The χ^2^ test was used to compare the number of patients in AMD and control groups. The Spearman rank correlation coefficient was calculated, and its significance was assessed. The odds ratios with 95% confidence intervals were calculated by logistic regression.

A logistic regression model adjusted for sex, age, living environment, and the CC genotype of the rs1061170 polymorphism was used to test the association between these parameters and AMD. *p*-values < 0.05 were considered statistically significant. The statistical analysis was performed using the STATISTICA 10.0 software.

## 3. Results

### 3.1. Characteristics of Patients and Controls

The characteristics of patients with AMD and controls showed significant differences in terms of living environment and family history of AMD among these groups. (Table 1). Female sex, age over 60 years, urban environment, and a positive family history of AMD were associated with a higher risk of AMD. The strongest positive correlation was observed for ages above 60 years and a positive family history (Table 2). Importantly, there was no correlation between the risk of AMD and smoking, which is considered to be one of the most important modifiable risk factors for AMD (Table 2).

### 3.2. Genotype and Allele Frequencies of Polymorphisms in Complement System Genes

The distribution of the genotypes and alleles of the SNPs in the CFH gene (Y402H rs1061170), C2 gene (E318D rs9332739), and C3 gene (R102G rs2230199) in patients with AMD and controls, as well as the results of the odds ratios (ORs) analysis, are shown in Table 3. The results showed strong evidence of an association between the CFH variant and AMD. For the SNP Y402H rs1061170, there were significant differences in the distribution of the CC (*p* = 0.0058) and TT (*p* = 0.0189) genotypes as well as C (*p* = 0.0311) and T (*p* = 0.0213) alleles between patients with AMD and controls (Table 3). The CC genotype and C allele were associated with a higher risk of exudative AMD (3.15 and 2.98 times higher, respectively), while the TT genotype and T allele were shown to protect against AMD (Table 3).

There were no differences between groups in the genotype and allele distribution for the SNPs E318D rs9332739 and R102G rs2230199 (Table 3).

The final logistic regression model included the following independent variables: sex, age, living environment, family history, and the CC genotype for the SNP rs1061170. The χ2 significance of the model was 31.463 and a *p*-value of 0.0000. The detailed results of the logistic regression analysis are presented in Table 4.

Model of logistic regression analysis (logit model):

$$\begin{array}{c} P\left(Y=AMD\right)\\ =\frac{e^{-5.5602+0.7683\times sex+1.7599\times age+0.8421\times living\;environment+1.6554\times family\;history+1.1561\times genotype\;CCrs1061170}}{1+e^{-5.5602+0.7683\times sex+1.7599\times age+0.8421\times living\;environment+1.1561\times genotype\;CCrs1061170}\;} \end{array}$$

SE, standard error; OR, odds ratio; CI, confidence interval.

### 3.3. Associations between Polymorphisms of Complement System Genes and Response to Anti-VEGF Therapy

The first-line treatment with bevacizumab was administered in 43 patients (38.7%) with AMD, while ranibizumab was administered in 68 patients (61.3%). In 28 cases (25.2%), there was no positive response to antiangiogenic therapy. These patients showed no improvement in eye function or reduction in CRT at 4 to 6 months compared with baseline.

Patients resistant to the first-line anti-VEGF drug were switched to the other anti-VEGF drug 4 to 6 months after initiation of the first-line therapy. Of the 28 poor responders, 16 were switched from ranibizumab to bevacizumab, and 12 from bevacizumab to ranibizumab. A follow-up assessment showed short-term benefits (from 2 to 4 months) in terms of improved BCVA and CRT in 17 poor responders (60.7%), while in 11 (39.3%) no anatomical and functional improvement was observed at 12 months. Finally, at the end of follow-up, all 28 poor responders showed worse BCVA (*p* = 0.0455) and greater CRT (*p* = 0.0328), as compared with individuals with a positive response to the first-line anti-VEGF therapy.

We observed an association between response to antiangiogenic therapy and the presence of selected genotypes for the SNP rs1061170 in the CFH gene in patients with AMD. We assessed the OR of poor response to therapy in patients with the CC and TT genotypes in comparison with those without these genotypes. The analysis showed that the CC genotype was associated with a higher risk of a negative response to antiangiogenic therapy (OR 8.75; 95% CI, 1.65–35.13; *p* = 0.0024) compared with patients with the CC and CT genotypes. On the other hand, the TT genotype was associated with a positive response to intravitreal anti-VEGF therapy (OR 0.29; 95% CI, 0.10–0.78; *p* = 0.0256), as compared with patients with the CC and CT genotypes. This association was also observed after switching to the other anti-VEGF drug. In our study group, 18 of the 28 poor responders (64.3%) were carriers of the CC genotype for the SNP rs1061170 in the CFH gene, while in the group of good responders, this genotype was detected only in 20 patients (24.1%; *p* = 0.0002) (Table 5). On the other hand, the TT genotype for the SNP rs1061170 in the CFH gene was present in 34.5% of good responders, as compared with 3.6% (1 patient) in the group of poor responders (*p* = 0.0001) (Table 5).

**Figure 1:**
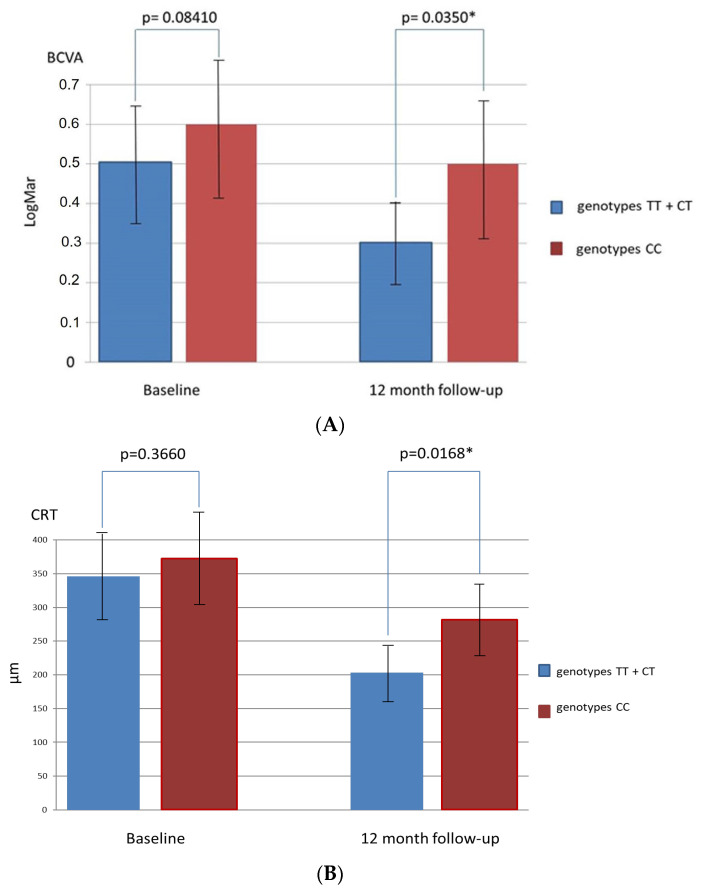
Changes in best corrected visual acuity (BCVA) (**A**) and central retinal thickness (CRT) (**B**) in patients with TT and CT genotypes for single nucleotide polymorphism rs1061170 of the CFH gene in comparison with patients with the CC genotype (* significant *p*-value < 0.05).

**Figure 2:**
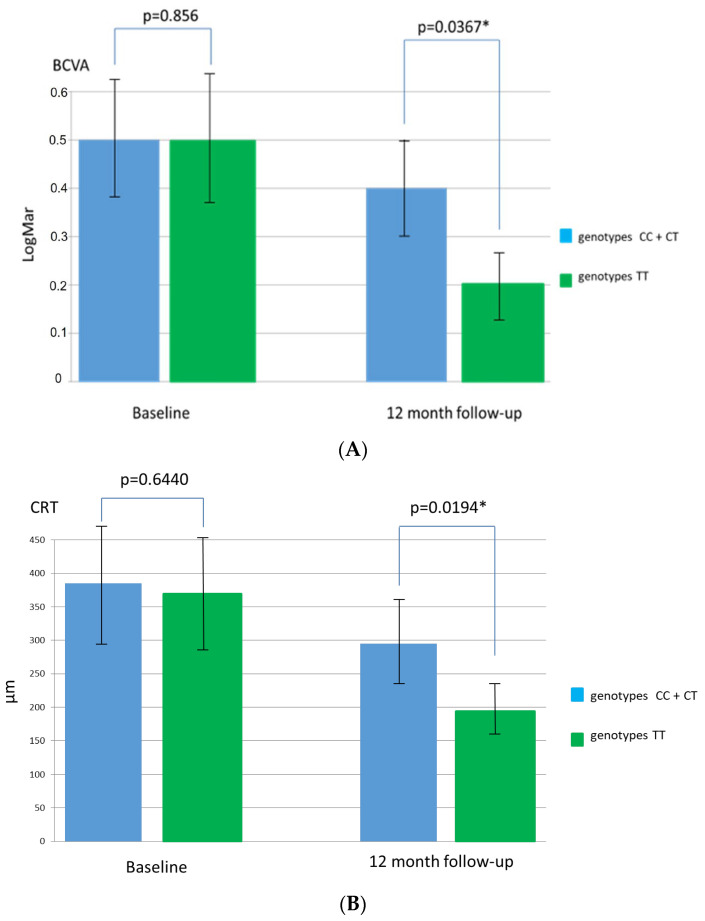
Changes in best corrected visual acuity (BCVA) (**A**) and central retinal thickness (CRT) (**B**) in patients with CC and CT genotypes for single nucleotide polymorphism rs1061170 of the CFH gene in comparison with patients with the TT genotype (* significant *p*-value < 0.05).

Changes in BCVA and CRT during follow-up in patients with TT + CT genotypes and CC genotype for the SNP rs1061170 in comparison with patients without these gen types are presented in Figure 1A,B and Figure 2A,B.

Our study also showed that patients with the CC genotype received a 1.5-fold higher mean number of intravitreal injections than patients without this genotype within a 12-month follow-up. Individuals with CC genotype for the CFH SNP rs1061170 received from 9 to 12 intravitreal injections (mean: 10.8), while patients with TT and CT genotypes—from 6 to 10 (mean: 7.2), and this difference was statistically significant (*p* = 0.0038). Another finding of the study was that irrespective of the presence of the SNP Y402H rs1061170 in the CFH gene, female sex, age above 60 years, urban environment, and positive family history were predictors of AMD.

There were no significant differences in age, sex, OCT findings, or CNV type and diameter between good and poor responders at baseline. However, the presence of serous PED at baseline was associated with a poor response as determined by both BCVA (OR 16.2; 95% CI, 2.56–33.2; *p* = 0.0221) and OCT findings (OR 23.0; 95% CI 1.80–61.5; *p* = 0.030) at 12 months. The baseline characteristics of good and poor responders, including the genotype frequencies for the SNP rs1061170 are presented in Table 5. 

## 4. Discussion

Recent studies have shown that the genetic factors may significantly affect the outcomes of antioxidant treatment, photodynamic therapy, and intravitreal anti-VEGF injections in patients with exudative AMD [45,46,47]. Most investigators emphasize the importance of the several SNPs of genes encoding complement proteins, particularly the SNP rs1061170 (Y402H) of the *CFH* gene [45,46,47,48,49,50,51,52,53,54,55,56,57].

To our knowledge, this is the first study to determine whether the variants of complement system genes affect response not only to the primary anti-VEGF therapy but also to treatment after switching to another drug in poor responders.

Based on a morphologic and functional analysis reported in the literature, up to 30% of AMD patients show poor response to anti-VEGF therapy [42,43,44]. In poor responders, disease progression occurs with an increase in CRT and deterioration of BCVA, ultimately leading to irreversible damage to central vision. The response to anti-VEGF therapy was found to depend on various factors including patient age, disease duration, baseline BCVA, and presence of risk alleles and genotypes. Some anatomical findings also seem to predict therapy failure, including subfoveal fibrosis, RPE and photoreceptor atrophy, presence of large lesions, type 1 CNV, serous, hemorrhagic, and fibrovascular PED, polypoidal choroidal vasculopathy, foveal scarring, and vitreomacular traction, outer retinal tubulation, and cystoid degeneration in the outer retina [35,38,41,42,43,44,58,59,60,61]. In our study, 25.2% of patients demonstrated poor response to anti-VEGF therapy and the presence of serous PED was the main anatomical predictor of poor response.

CFH is an important regulator of the alternative pathway of the complement system. Its genetic polymorphism Y402H (rs1061170) (tyrosine-to-histidine transition) causes the hyperactivation of the alternative complement pathway resulting in cell damage, including RPE cells [35]. Immunohistochemical studies showed that homozygous carriers of the CC genotype have a 2.5-fold stronger immune reaction to C-reactive protein (CRP) in the choroid, Bruch’s membrane, and drusen [46,50]. The CRP accumulation under the RPE is considered a marker of chronic inflammation in the RPE-choroid complex [50]. This suggests that the SNP rs1061170 in the *CFH* gene impairs the H factor-mediated CRP function, thus playing an important role in inducing local inflammation that leads to RPE damage caused by complement activation. The SNP rs1061170 in the *CFH* gene reduces or completely impairs the protective function of factor H, which suppresses the activation of the alternative complement pathway both in plasma and the inflamed tissue [33]. Moreover, in patients with the presence of environmental and individual risk factors for AMD, the polymorphism reduces the activity of CFH as a regulator of the alternative complement pathway, thus promoting its uncontrolled activation. This leads to the development of chronic local inflammation (parainflammation or inflammaging), resulting in macular lesions [33,50]. Throughout life, the retina is subject to oxidative damage, which affects the expression of complement components in the aging retina and AMD-diseased tissues [62,63,64,65,66]. The oxidized photoreceptor outer segment material was shown to reduce the synthesis of complement factor H in cultured RPE cells [61]. This is consistent with the findings of reduced levels of factor H in Bruch’s membrane, choriocapillaris, and the choroid of AMD specimens compared with controls [65].

The involvement of proinflammatory mechanisms in AMD is supported also by the presence of complement component proteins in drusen and choroidal neovascular membranes in patients with AMD [64]. Moreover, patients with AMD were shown to have higher serum levels of complement proteins C2 and C3 [67]. Recently, Cipriani et al. reported the association between increased circulating levels of factor H-related protein 4 and AMD [66].

Interestingly, anti-VEGF therapy for exudative AMD may enhance local complement activation. Experimental studies showed that CFH synthesized by RPE cells is protective against complement-mediated damage, as it is considered the most important inhibitor of the alternative complement pathway [67,68]. CFH production is upregulated by VEGF, and the loss of RPE-derived VEGF reduces CFH expression. This makes the outer retina vulnerable to complement-mediated inflammation, the activation of which is one of the most crucial factors involved in the pathogenesis of AMD. Reduced CFH expression in the outer retina and local complement activation were also observed in enucleated AMD donor eyes as compared with controls [68]. Several studies showed that RPE cells from AMD patients carrying AMD-associated *CFH* 402H polymorphism had more C3d deposits compared with a control group. This suggests an increased activation of the alternative pathway on the surface of AMD RPE cells that possess this polymorphism [68,69]. Of note, Keir et al. [68] suggested that VEGF inhibitors could exacerbate local complement activation by reducing CFH synthesis. Moreover, this was more pronounced in RPE cells expressing *CFH* Y402H, possibly because they already have a reduced complement regulatory capacity and anti-VEGF treatment could result in a further decrease. This phenomenon could explain why patients with the *CFH* 402H polymorphism, especially homozygosity, are at greater risk of AMD and may demonstrate a poorer response to anti-VEGF therapy.

In addition, epidemiological studies revealed that the presence of the SNP rs1061170 in the *CFH* gene is associated with a 2- to 4-fold higher risk of AMD in heterozygous carriers and a 3- to 7-fold higher risk in homozygous carriers [49]. Moreover, carriers of the homozygous CC genotype showed a worse response to oral antioxidant and zinc treatment compared with carriers of the homozygous TT genotype [45].

Numerous studies reported an association between the *CFH* rs1061170 polymorphism and response to local antiangiogenic therapy [31,32,33,34,35,51,52,53,54,55]. Brantely et al. [31] investigated patients treated with intravitreal bevacizumab. They showed that carriers of the CC genotype for the SNP rs1061170 in the *CFH* gene had worse BCVA at 6 months than TT and CT genotype carriers [31]. This is in line with studies by Nischler et al. and Imai et al. [34,35]. On the other hand, Lee et al. [33] reported that the genotypes of this SNP were not associated with BCVA in patients with exudative AMD treated with intravitreal ranibizumab injections. However, at 9 months, patients with the CC genotype received approximately 1 more injection than patients with the TT or CT genotypes [33]. Mckibbin et al. [55] showed better treatment outcomes at 6 months in CC genotype carriers with exudative AMD treated with ranibizumab. Menghini et al. [56] revealed that the CT genotype for this SNP was a significant predictor of a positive response to treatment and improvement in BCVA at 12 and 24 months of ranibizumab treatment. On the other hand, interesting results were reported by the CATT trial (Comparison of AMD Treatments Trials) including patients from 43 centers, who were tested for the presence of SNP rs1061170 in the *CFH* gene and rs2230199 in the *C3* gene in addition to rs10490924 (ARMS2) and rs11200638 (HTRA1). The analysis did not reveal any differences in response to local antiangiogenic therapy between carriers of different genotypes. None of the tested genotypes showed a significant effect on vision acuity, changes in CRT, or the number of intravitreal anti-VEGF injections. Moreover, there were no associations between the number of AMD risk genotypes and treatment outcomes. No associations were also shown between the presence of AMD risk alleles or genotypes and response to treatment, irrespective of the type of intravitreal drug or treatment regimen (1 injection per month vs. as needed) [70].

However, in line with most studies, our results indicated a significant correlation between the SNP rs1061170 in the *CFH* gene and the risk of AMD [20,21,22,23,24,25,26,27,28,29,30]. During the 12-month follow-up, CC genotype carriers showed worse functional and anatomical outcomes of intravitreal ranibizumab and bevacizumab injections, as assessed on the basis of BCVA and CRT, than other genotype carriers. At 12 months, they received approximately 1.5 injections more than patients without this genotype. This observation may suggest that the CC genotype for the SNP rs1061170 in the *CFH* gene may promote a poor response to anti-VEGF therapy, independently of the anti-VEGF agent used. Thus, the rs1061170 variant of the *CFH* gene may predict the clinical course of the disease and may help assess the need for additional injections.

However, except for the rs1061170 variant of the *CFH* gene, other AMD gene polymorphisms and other factors may play a role in the poor reaction to anti-VEGF therapy.

Inadequate response to antiangiogenic therapy in patients with exudative AMD may also be caused by tachyphylaxis, which may occur in up to 10% of cases [71,72,73,74]. In tachyphylaxis, repeated drug administration leads to a noticeable weakening of the effect and symptom recurrence despite a positive initial response to treatment. The timing of tachyphylaxis onset remains unclear and possibly depends on individual factors. The most effective method for the prevention of tachyphylaxis is to replace one drug with another from the same class [71,72]. However, some authors indicated that the potential mechanism underlying a reduced response to anti-VEGF therapy is associated with resistance rather than with tachyphylaxis, and this mechanism remains unclear in exudative AMD [74]. In addition, alteration in CNV architecture due to chronic inflammation and fibrosis which acts as a resorption barrier may decrease sensitivity to anti-VEGF drugs [71].

Our results showed that the SNPs E318D rs9332739 in the *C2* gene and R102G rs2230199 in the *C3* gene are not associated with the risk of AMD. Spencer et al. [75] revealed that the SNP E318D protects against AMD in the white population in the United States. However, these findings were not corroborated by an Australian study [76]. Similarly, Havvas et al. [77], in a study on 120 Greek patients with AMD, did not show this polymorphism to be implicated in the pathogenesis of AMD. Its protective role was not confirmed in a study on an Asian population with a low prevalence of this SNP [78]. On the other hand, the protective role of the SNP R102G rs2230199 in the *C3* gene was shown in Asian patients in studies by Pei et al. and Yanagisawa et al. [79,80]. However, these findings were not corroborated by a study on a German population [81]. The discrepancies between studies can be explained by ethnic differences and environmental factors [82]. In addition, the cumulative effect of the numerous AMD risk alleles or genotypes on the prognosis of response to antiangiogenic therapy cannot be excluded [54]. Therefore, to improve the prognostication of treatment failure, there is an ongoing search for other genetic variants associated with the clinical course and treatment of AMD [57].

This study has some limitations. The sample size was relatively small, and the genetic analysis was limited to several genetic variants of the complement system. Additionally, this was a retrospective study and we included individuals treated only with two anti-VEGF drugs (ranibizumab and bevacizumab). Research on a larger group of patients treated also with other VEGF inhibitors (aflibercept and brolucizumab) as well as the analysis of other genetic polymorphisms of the complement system is warranted to validate our observations.

## 5. Conclusions

In conclusion, we found that the CC genotype for the *CFH* SNP rs1061170 was associated with AMD and promoted a poor response to therapy, independently of the type of the VEGF inhibitor used. On the other hand, the TT genotype protected against AMD and was associated with significantly better functional and anatomical outcomes compared with individuals without this genotype. This correlation was also observed after switching to the other VEGF inhibitor. The CC genotype for the *CFH* SNP rs1061170 was also associated with a greater number of additional anti-VEGF intravitreal injections during the 12-month follow-up.

## Figures and Tables

**Table 1:** Characteristics of patients with age-related macular degeneration (AMD) and controls.

Parameter		AMD Group (*n* = 111)	Control Group (*n* = 58)	*p*-Value
Sex				0.0624
	Female	73 (65.8)	37 (63.8)	
	Male	38 (34.2)	21 (36.2)	
Age, y, range (avereage)		56–90 (71.3)	54–88 (69.8)	0.0732
	≤60 years	16 (14.4)	11 (19.0)	
	>60 years	95 (85.6)	47 (81.0)	
Smoking				0.0796
	Current or former	34 (30.6)	15 (25.8)	
	Never	77 (69.4)	43 (74.2)	
Living environment				0.0158 ^1^
	Urban	77 (69.4)	36 (62.0)	
	Rural	34 (30.6)	22 (38.0)	
Family history of AMD				0.0021 ^1^
	Positive	87 (78.4)	4 (4.0)	
	Negative	24 (21.6)	54 (96.0)	

Categorial variables are presented as numbers (percentages). ^1^ significant *p*-value (<0.05).

**Table 2:** Risk of age-related macular degeneration (AMD) associated with sex, age, smoking, living Environment, and family history of AMD.

Parameter		AMD Group (*n* = 111)	Control Group (*n* = 58)	Adjusted OR (95% CI)	*p*-Value for *n* (%) Compared
Sex	Males	38 (34.2)	21 (36.2)	Ref.	0.0282 ^1^
	Females	73 (65.8)	37 (63.8)	2.12 (1.09–4.16)	
Age	≤60 years	16 (14.4)	11 (19.0)	Ref.	0.0002 ^1^
	>60 years	95 (85.6)	47 (81.0)	5.56 (2.23–13.86)	
Smoking	Current or former	34 (30.6)	15 (25.8)	0.50 (0.13–1.24)	0.0831
	Never	77 (69.4)	43 (74.2)	Ref.	
Living environment	Urban	77 (69.4)	36 (62.0)	2.65 (1.18–4.95)	0.0191 ^1^
	Rural	34 (30.6)	22 (38.0)	Ref.	
Family history of AMD	Positive	87 (78.4)	4 (4.0)	8.56 (3.64–14.80)	0.0023 ^1^
	Negative	24 (21.6)	54 (96.0)	Ref.	

Categorical variables are presented as numbers (percentages). OR—odds ratio; CI—confidence interval; Ref—the reference group. ^1^ significant *p*-value (<0.05).

**Table 3:** Genotype and allele frequencies of polymorphisms in the *CFH*, *C2*, and *C3* genes in patients with age-related macular degeneration (AMD) and controls.

Polymorphism	Genotype/Allele	AMD Group (*n* = 111)	Control Group (*n* = 58)	Adjusted OR (95% CI)	*p*-Value for *n* (%) Compared
Y402H rs1061170 (*CFH*)	TT	21 (19.0)	19 (33.0)	Ref.	
	CC	38 (34.2)	9 (15.0)	3.15 (1.24–7.66)	0.0058 ^1^
	CT	52 (46.8)	30 (52.0)	2.52 (1.41–5.68)	0.5422
	T	94 (52.3)	68 (58.6)	Ref.	
	C	128 (57.7)	48 (41.4)	3.98 (1.32–8.47)	0.0311 ^1^
E318D rs9332739 (*C2*)	GG	104 (93.7)	51 (88.0)	Ref.	
	gc	7 (6.3)	7 (12.0)	0.86 (0.25–1.64)	0.1154
	G	208 (93.6)	102 (88.0)	Ref.	
	g	7 (3.2)	7 (6.0)	0.53 (0.11–1.24)	0.2330
	c	7 (3.2)	7 (6.0)	0.53 (0.11–1.22)	0.2330
R102G rs2230199 (*C3*)	GG	68 (61.3)	40 (69.0)	Ref.	
	gc	33 (29.7)	16 (28.0)	2.15 (0.43–10.88)	0.4650
	cc	10 (9.0)	2 (3.0)	1.38 (0.62–4.95)	0.2845
	G	136 (61.2)	80 (69.0)	Ref.	
	g	33 (14.9)	16 (14.0)	1.10 (0.85–2.31)	0.3622
	c	53 (23.9)	20 (17.0)	1.68 (1.17–2.24)	0.5542

Categorical variables are presented as numbers (percentages). OR—odds ratio; CI—confidence interval; Ref.—the reference group. ORs were adjusted for sex and age. ^1^ significant *p*-value (<0.05).

**Table 4:** Results of logistic regression analysis based on sex, age, living environment, and the CC genotype for the rs1061170 polymorphism.

Variable	Estimated Parameter Value	SE	95% CI for Estimated Parameter Value		Wald Test		OR	95% CI for OR	
			Lower	Upper	χ^2^	*p*-Value		Lower	Upper
Coefficient β0	−5.5602	1.3962	−8.3177	−2.8027	15.8596	0.0001	0.004	0.000	0.061
Sex	0.7683	0.3690	0.0396	1.4970	4.3356	0.0373	2.156	1.040	4.468
Age	1.7599	0.5076	0.7573	2.7625	12.0193	0.0005	5.812	2.133	15.839
Living environment	0.8421	0.4001	0.0519	1.6322	4.4303	0.0353	2.321	1.053	5.115
Family history	1.6554	0.4761	0.6545	2.3886	10.332	0.0033	8.312	3.630	14.043
CC genotype for rs1061170	1.1561	0.4480	0.2713	2.0409	6.6587	0.0099	3.178	1.312	7.698

**Table 5:** Baseline characteristics of patients with age-related macular degeneration (AMD) according to response to anti-VEGF therapy.

Parameter		Good Responders	Poor Responders	*p*-Value
No. of patients		83 (74.8)	28 (25.2)	0.0001 ^1^
Age, years, range (average)		56–88 (70.9)	58–90 (71.6)	0.8655
First-line anti-VEGF drug number of eyes (%)	Bevacizumab	31 (37.3)	12 (42.9)	0.0633
	Ranibizumab	52 (62.7)	16 (57.1)	0.0976
Baseline BCVA [LogMAR], range (mean)		1.5–0.4 (0.5)	1.4–0.4 (0.5)	0.0678
Baseline CRT (µm)		188–605 (342.6)	201–614 (361.1)	0.4568
Presence of IRF		68 (82.0)	22 (78.6)	0.4590
Presence of SRF		39 (47.0)	11 (39.3)	0.0853
Presence of sPED		23 (27.7)	15 (53.6)	0.0057 ^1^
Baseline CNV area on FA (mm^2^), range (average)		1.2–3.4 (2.24)	1.1–3.2(2.48)	0.0830
Genotypes for the *CFH* gene polymorphism	CC CT TT	20 (24.1) 40 (48.2) 20 (34.5)	18 (64.3) 9 (32.1) 1 (3.6)	0.0002 ^1^ 0.0830 0.0001 ^1^

Categorical variables are presented as numbers (percentages). ^1^ Significant *p*-value (<0.05); BCVA—best-corrected visual acuity; CRT—central retinal thickness, sPED—serous pigment epithelium detachment; SRF—subretinal fluid; IRF—intraretinal fluid; CNV—choroidal neovascularization; FA—fluorescein angiography.

## Data Availability

All the data are available from the corresponding author upon reasonable request.
